# Supplementary material for: Hybrid Models and Biological Model Reduction with PyDSTool
Source: PLoS Comput Biol. 2012 Aug 9;8(8):e1002628. doi: 10.1371/journal.pcbi.1002628 (PMC3415397; doi:10.1371/journal.pcbi.1002628)
Supplement: Text S4 — Complete source code for the PyDSTool package (version 0.88.120504). Includes API documentation and help files linking to web pages. This file is identical to the current public release on Sourceforge.net. (ZIP) [file pcbi.1002628.s004.zip › PyDSTool/html/PyDSTool.Toolbox.optimizers.step.marquardt_step-pysrc.html]

xml version="1.0" encoding="ascii"?


PyDSTool.Toolbox.optimizers.step.marquardt\_step


| Home | Trees | Indices | Help | PyDSTool |
| --- | --- | --- | --- | --- |

Package PyDSTool ::
Package Toolbox ::
Package optimizers ::
Package step ::
Module marquardt\_step

[hide private]

[frames] | no frames]

# Source Code for Module PyDSTool.Toolbox.optimizers.step.marquardt\_step

```
 1   
 2  # Matthieu Brucher 
 3  # Last Change : 2007-08-29 13:42 
 4   
 5  """ 
 6  Computes a Marquardt step for a specific function at a specific point 
 7  """ 
 8   
 9  import numpy 
10  import numpy.linalg 
11   


12 -class MarquardtStep(object):


13    """ 
14    The simple gradient step 
15    """ 


16 -  def __init__(self, **kwargs):


17      """ 
18      Computes the Marquardt step 
19        - gamma is the initial Marquardt correction factor (gamma = 1e4) 
20        - c1 is the decreasing factor (c1 = 0.5) 
21        - c2 is the increasing factor (c2 = 2.) 
22      """ 
23      self.gamma = kwargs.get('gamma', 1.e4) 
24      self.c1 = kwargs.get('c1', 0.5) 
25      self.c2 = kwargs.get('c2', 2.)

26   


27 -  def __call__(self, function, point, state):


28      """ 
29      Computes a gradient step based on a function and a point 
30      """ 
31      if 'gamma' in state: 
32        gamma = state['gamma'] 
33      else: 
34        gamma = self.gamma 
35   
36      hessian = function.hessian(point) 
37      gradient = function.gradient(point) 
38      approx_hessian = hessian + numpy.diag([gamma] * len(gradient)) 
39      step = (-numpy.linalg.solve(approx_hessian, gradient)).reshape(point.shape) 
40   
41      f0 = function(point) 
42      f1 = function(point + step) 
43      if f1 < f0: 
44        gamma = self.c1 * gamma 
45      else: 
46        gamma = self.c2 * gamma 
47   
48      state['hessian'] = hessian 
49      state['approx_hessian'] = approx_hessian 
50      state['gradient'] = gradient 
51      state['direction'] = step 
52      state['gamma'] = gamma 
53      return step

54
```

  


| Home | Trees | Indices | Help | PyDSTool |
| --- | --- | --- | --- | --- |

|  |  |
| --- | --- |
| Generated by Epydoc 3.0.1 on Fri May 4 15:24:12 2012 | http://epydoc.sourceforge.net |
